# DNA damage, demethylation and anticancer activity of DNA methyltransferase (DNMT) inhibitors

**DOI:** 10.1038/s41598-023-32509-4

**Published:** 2023-04-12

**Authors:** Angelo B. A. Laranjeira, Melinda G. Hollingshead, Dat Nguyen, Robert J. Kinders, James H. Doroshow, Sherry X. Yang

**Affiliations:** 1 Division of Cancer Treatment and Diagnosis, National Cancer Institute, Bethesda, MD USA; 2 Leidos Biomedical Research, Inc., Frederick, MD USA; 3 Division of Cancer Treatment and Diagnosis, National Clinical Target Validation Laboratory, 9609 Medical Center Drive, Bethesda, MD 20892 USA

**Keywords:** Cancer, Molecular biology

## Abstract

Role of DNA damage and demethylation on anticancer activity of DNA methyltransferase inhibitors (DNMTi) remains undefined. We report the effects of *DNMT1* gene deletion/disruption (*DNMT1*^*−/−*^) on anticancer activity of a class of DNMTi in vitro, in vivo and in human cancers. The gene deletion markedly attenuated cytotoxicity and growth inhibition mediated by decitabine, azacitidine and 5-aza-4′-thio-2′-deoxycytidine (aza-T-dCyd) in colon and breast cancer cells. The drugs induced DNA damage that concurred with DNMT1 inhibition, subsequent G_2_/M cell-cycle arrest and apoptosis, and upregulated p21 in *DNMT1*^+*/*+^ versus *DNMT1*^*−/−*^ status, with aza-T-dCyd the most potent. Tumor growth and DNMT1 were significantly inhibited, and p21 was upmodulated in mice bearing HCT116 DNMT1^+/+^ xenograft and bladder PDX tumors. *DNMT1* gene deletion occurred in ~ 9% human colon cancers and other cancer types at varying degrees. Decitabine and azacitidine demethylated *CDKN2A*/*CDKN2B* genes in *DNMT1*^+*/*+^ and *DNMT1*^*−/−*^ conditions and increased histone-H3 acetylation with re-expression of p16^INK4A^/p15^INK4B^ in *DNMT1*^*−/−*^ state. Thus, *DNMT1* deletion confers resistance to DNMTi, and their anti-cancer activity is determined by DNA damage effects. Patients with *DNMT1* gene deletions may not respond to DNMTi treatment.

## Introduction

DNA methylation is essential for mammalian development and differentiation and a hallmark of tumorigenesis^1–3^. DNA methyltransferase (DNMT) 1 is an enzyme functional for maintenance and propagation of DNA methylation pattern in proliferating cells^4,5^. It adds a methyl group in carbon 5 (C5) position of cytosine to produce C5-methyl-cytosine in the context of CpG dinucleotide sequences. DNMT1 is implicated in silencing tumor suppressor genes not only through methylation of the CpG sites but also via participating in the formation of transcriptionally repressive complex^6^. Inhibition of DNMT1 reduces the levels of DNA methylation and re-activates tumor suppressor genes e.g., *CDKN2A* (Chromosome 9, band p21.3) and *CDKN2B* juxtaposition to *CDKN2A* either with knocking out of *DNMT1* or in combination with histone deacetylase inhibition^7,8^.

Decitabine (5-aza-2′-deoxycytidine or DAC) and azacitidine (5-azacytidine or AZA) are prototype cytidine analogs, known as DNMT inhibitors (DNMTi) or hypomethylating agents^9,10^. Both are approved for the treatment of myelodysplastic syndrome and acute myeloid leukemia. 5-Aza-4′-thio-2′-deoxycytidine (aza-T-dCyd or aza-TdC), a novel anti-DNMT agent, inhibits DNMT1 in vitro and in vivo^11–13^. The incorporation of decitabine and azacitidine into DNA led to their covalent binding to DNMT1^14^, which blocks DNA methyltransferase function and demethylates CpG sites upon DNA replication^15^. The formation of DNA-DNMT protein adducts triggers DNA damage and elicits DNA damage response^16–18^. Overall, the reactivation of the silenced tumor suppressor genes through CpG demethylation as an epigenetic mechanism was proposed as a mode of anticancer activity of DNMTi although its clinical translation has met considerable difficulties, particularly in solid tumors^19–21^. A close and robust connection between demethylation and reactivation of tumor suppressors and clinical efficacy is not established in human malignancies^22^. Therefore, elucidating the mechanisms of anticancer activity of DNMTi warrants both genetic and epigenetic means of investigation.

Using the models with intact and targeted deletion or disruption of *DNMT1* gene alleles, in this study, we addressed the mechanisms of anti-cancer activity of DNMTi that are mediated (1) primarily by epigenetic mechanism of demethylation and reactivation of expression of tumor suppressor genes; (2) largely through DNA damage effects; and (3) via both modes of action. We show that a class of anti-DNMT agents including decitabine, azacitidine and aza-T-dCyd inhibited DNMT1 expression and induced the DNA damage effects at varying degrees in *DNMT1*^+*/*+^ HCT116 and MCF7 cells. The higher anticancer activity—cell cycle arrest, apoptosis and cell death—of aza-T-dCyd was proportional to its greater degree of induction of DNA damage without activating p16^INK4A^ and p15^INK4B^. By contrast, re-expression of the tumor suppressors coupled to the CpG demethylation and enhanced acetylation of histone H3 at the gene loci corresponded to a lack of apoptosis and cell death by decitabine and azacitidine in *DNMT1* gene deleted/disrupted status. We also investigated *DNMT1* gene deletion in human cancers.

## Results

### Deletion or disruption of *DNMT1* gene confers resistance to DNMTi in cancer cells

**Figure 1:**
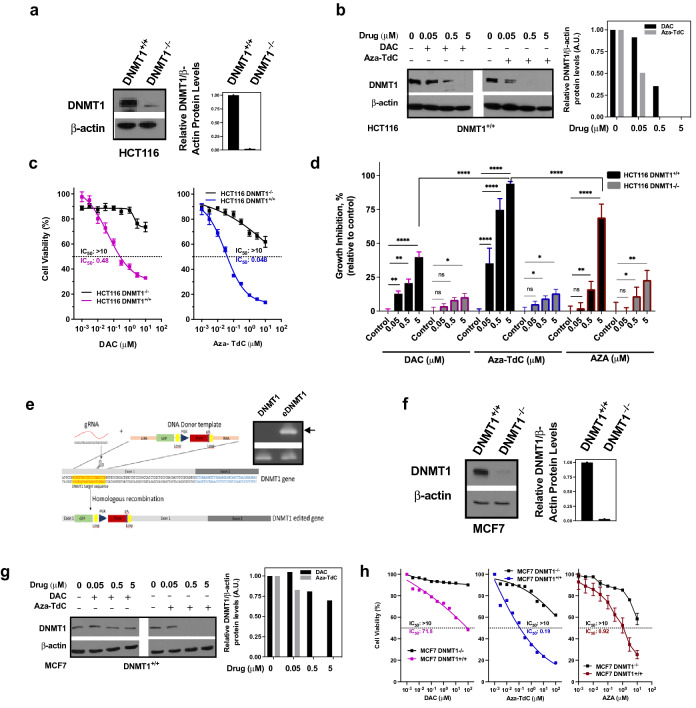
Effects of DNMTi on DNMT1, cytotoxicity and growth in models with intact and disrupted *DNMT1* gene. (**a**) Western blotting analysis and quantitation of DNMT1 in HCT116 DNMT1^+/+^ and DNMT1^−/−^ cells. (**b**) Inhibition of DNMT1 following exposure to DAC and aza-T-dCyd for 48 h in HCT116 DNMT1^+/+^ cells. (**c**) Cytotoxicity of DAC and aza-T-dCyd after treatment for 96 h in the HCT116 pair. (**d**) Colony formation inhibition of HCT116 pair by three inhibitors as indicated. (**e**) Scheme of disruption of *DNMT1* gene using CRISPR technology in MCF7 cells. Inset: PCR confirmation of genomic integration of the donor template DNA (arrow: 379 bp insertion, eDNMT1) into the *DNMT1* gene relative to the scrambled control. (**f**) DNMT1 phenotype in the MCF7 pair probed with DNMT1 antibody. (**g**) Inhibition of DNMT1 protein by DAC and aza-T-dCyd in MCF7 DNMT1^+/+^ cells after 48 h of drug exposure. (**h**) Cytotoxicity of DAC, 5-aza-T-dCyd and AZA in the MCF7 cell pair. All samples in the cytotoxicity and colony formation experiments were assayed in triplicate, with at least three independent experiments performed, and data were shown as mean ± SD. *P < 0.05; **P < 0.01; ****P < 0.0001; and ns, not significant. One representative experiment is shown from at least three independent experiments for other assays.

DNMT1 phenotype in HCT116 *DNMT1*^+*/*+^ and *DNMT1*^*−/−*^ colorectal cancer cells were confirmed by probing with DNMT1 antibody (Fig. 1a and Supplementary Fig. 5a). DNMT1 was inhibited by decitabine and aza-T-dCyd as well as azacitidine in *DNMT1*^+*/*+^ cells (Fig. 1b, Supplementary Figs. 1a, 5b and 8a). Noticeably, there was a low level of DNMT1 expression in *DNMT1*^*−/−*^ cells. Cytotoxicity was examined in the DNMT1 cell models (Fig. 1c). IC50s of decitabine and aza-T-dCyd were 0.48 µM and 0.048 µM in DNMT1^+/+^ cells, respectively. With *DNMT1* deletion and knocking down of DNMT1 protein, the doses that inhibited a half of cancer cells were all higher than 10 µM. We subsequently examined clonogenic cell growth by increasing concentrations of DNMTi after 48 h of drug exposure. The average inhibition of growth was 39.9 ± 11.4%, 94.0% ± 5.1% and 69.2% ± 9.8% by 5 µM of decitabine, aza-T-dCyd and azacitidine, respectively (Fig. 1d); in contrast, about 25% or less of growth inhibition by all drugs in *DNMT1*^*−/−*^ models. Therefore, by all drugs in DNMT1^−/−^ models (Fig. 1d). Therefore, the *DNMT1* gene deletion caused dramatic increase in resistance to anti-DNMT agents. To validate the findings of HCT116–*DNMT1* model, *DNMT1* gene alleles in breast cancer MCF-7 cells was disrupted using CRISPR gene editing technology. The editing diagram and PCR confirmation of genomic integration of the donor template DNA into the *DNMT1* gene were shown in Fig. 1e; and the result of DNMT1 knockdown was confirmed by Western blotting (Fig. 1f and Supplementary Fig. 5f). DNMT1 expression was inhibited in MCF7 cells with intact DNMT1 gene by decitabine and aza-TdCyd (Fig. 1g and Supplementary Fig. 5g). The cytotoxicity (Fig. 1h) and inhibition of the colony formation by DNMTi (Supplementary Fig. 1b) were much greater in *DNMT1*^+*/*+^ than *DNMT1*^*−/−*^ cells. Taken together, the findings indicate that disruption of *DNMT1* gene and knocking down its protein expression confers resistance to DNMTi. Aza-T-dCyd is more potent than decitabine and/or azacitidine to inhibit DNMT1 and has higher cytotoxic and growth inhibitory effects.

### Coupling DNMT1 inhibition to DNA damage, and cell cycle arrest, apoptosis and cell death

**Figure 2:**
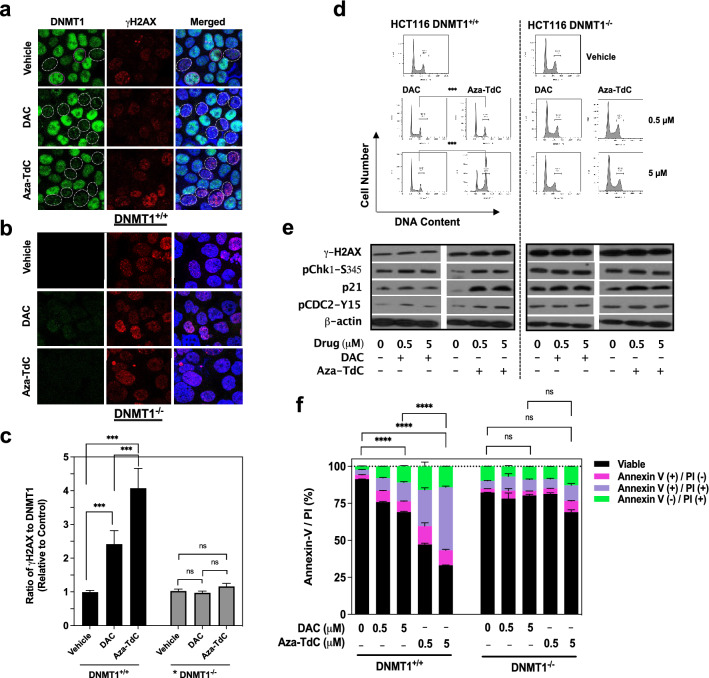
Effects of DNMTi on DNMT1 and DNA damage, cell-cycle, and apoptosis in HCT116 cells. (**a**) Confocal microscopy showing inhibition of DNMT1 (green fluorescence, white circles) and induction of γH2AX (red fluorescence) upon exposure to 5 µM of DAC and aza-TdC at 30 min in DNMT1^+/+^ nuclei (merged, white circles); intrinsic γH2AX and DNMT1 in vehicle-treated DNMT1^+/+^ cells (white and purple circles). (**b**) DNMT1 and γH2AX in DNMT1^−/−^ cells. (**c**) Ratio of γH2AX to DNMT1 intensity by DAC and aza-TdC. *Low level of DNMT1 expression was detected in DNMT1^−/−^ cells. (**d**) Cell-cycle distribution after exposure to DAC and aza-TdC for 24 h. (**e**) Activation of the cell-cycle checkpoint effectors by DAC and aza-TdC. (**f**) Induction of early apoptosis (annexin+/PI−), late apoptosis (annexin+/PI+) and cell death (annexin−/PI+) upon exposure to DAC and aza-TdC for 96 h. All samples in the cell-cycle and apoptosis experiments were assayed in triplicate, with at least three independent experiments performed, and data were shown as mean ± SD. ***P < 0.001; ****P < 0.0001; *ns* not significant. *PI* propidium iodide. One representative experiment is shown from at least three independent experiments for other assays.

DNMT1 was previously shown to be covalently trapped in decitabine-substituted DNA, leading to the DNA damage^14,16,18,23^. To determine the implication of aza-T-dCyd and decitabine in DNMT1 inhibition and DNA damage, we examined the induction of γH2AX (indicative of DNA stand breaks) and inhibition of DNMT1 upon exposure to both drugs for 30 min in HCT116-*DNMT1* model. γH2AX was induced in the nuclei in which DNMT1 was inhibited by the drugs, and little γH2AX induced where DNMT1 was not inhibited in DNMT1^+/+^ cells (Fig. 2a). The intensity of γH2AX signal was greater by aza-T-dCyd than that produced by decitabine. Intrinsic γH2AX was randomly distributed in vehicle treated DNMT1^+/+^ cells, irrelevant to the DNMT1 levels. In contrast, expression of γH2AX were not changed in the nuclei with the drug treatment in DNMT1^−/−^ cells (Fig. 2b). The ratios of γH2AX to DNMT1, relative to the control, was 2.3-fold and 4.1-fold by decitabine and aza-T-dCyd, respectively (P < 0.001; Fig. 2c). The ratio was constant in *DNMT1*^*−/−*^ cells regardless of drug exposure, indicating that DNMTi had no significant DNA damage effects in *DNMT1*^*−/−*^ cells. The results suggest that inhibition of DNMT1 is implicated in the DNA damage upon exposure to DNMTi and aza-T-dCyd is more potent than decitabine for such activity in the presence of DNMT1.

With DNMTi that caused differential DNA damages between DNMT1^+/+^ and DNMT1^−/−^ cells, we next investigated their effects on the cell-cycle by flow cytometry. Exposure to aza-T-dCyd for 24 h induced cell-cycle arrest at G_2_/M phase in HCT116 DNMT1^+/+^ versus DNMT1^−/−^ cells (Fig. 2d). The arrest by aza-T-dCyd was dose-dependent, which differed significantly from decitabine at both 0.5 µM and 5 µM dose levels (P < 0.001 each). The cell-cycle distribution was not altered as a function of drug exposure in the *DNMT1*^*−/−*^ cells. The cell-cycle arrest was also observed in MCF7 DNMT1^+/+^ versus DNMT1^−/−^ cells by decitabine, aza-T-dCyd and azacitidine (Supplementary Fig. 1c). We then examined the molecular effectors of the cell cycle distribution (Fig. 2e, Supplementary Table and Supplementary Fig. 6). Decitabine and aza-T-dCyd up-modulated γH2AX and p21, and activated checkpoint kinase 1 (pChk1-Ser345), with greater effects imposed by aza-T-dCyd. These were accompanied with an increase in pCDC2-Y15 and a near depletion of histone H3 phosphorylation (Supplementary Figs. 1d and 8d), indicating a main arrest at G_2_ phase in *DNMT1*^+*/*+^ cells.

We subsequently examined the apoptosis and cell death 96 h after treatment with decitabine and aza-T-dCyd using annexin-V/propidium iodide (PI) apoptosis assay in HCT116-*DNMT1* model (Fig. 2f). The apoptosis and cell death induced by aza-T-dCyd were ~ twofold greater than decitabine (*P* < 0.0001). In contrast, both drugs did not significantly induce apoptosis in *DNMT1*^*−/−*^ cells (*P* > 0.05 each). Apoptosis and cell death were also attained in MCF-7 *DNMT1*^+*/*+^ as opposed to *DNMT1*^*−/−*^ cells by azacitidine (Supplementary Fig. 1e). It is worth noting that the majority of MCF7 by azacitidine treatment are annexin-negative and PI-positive while HCT116 cells are annexin-positive and PI-positive treated with decitabine and aza-T-dCyd upon 96 h of treatment. The data indicate that substantial MCF7 cells underwent necrosis while most of HCT116 cells were in the late phase of apoptosis. In brief, DNMTi treatment leads to DNA damage, and mediates the cell-cycle arrest and apoptosis in *DNMT1*^+*/*+^ cells versus *DNMT1*^*−/−*^ cells.

### Demethylation, acetylation, and reexpression of p16^INK4A^ and p15^INK4B^ in ***DNMT1***^***−/−***^ versus ***DNMT1***^+***/***+^ status

**Figure 3:**
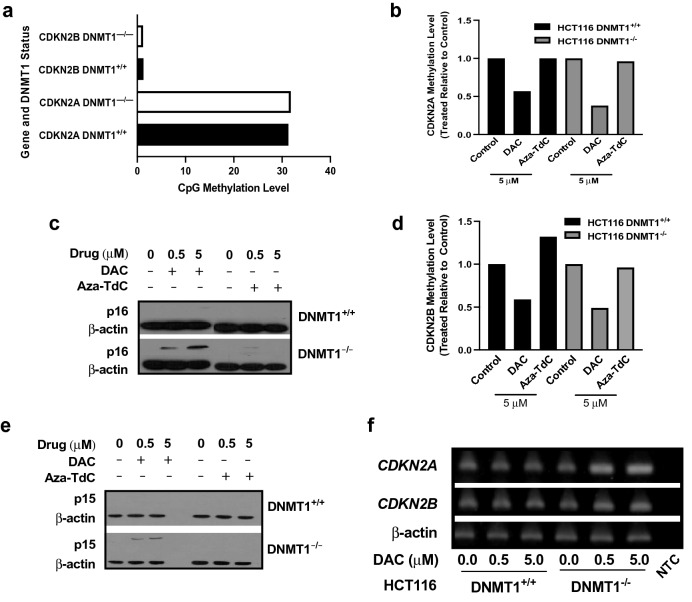
Demethylation of *CDKN2A* and *CDKN2B* genes, acetylation of histone H3, and re-expression of p16^INK4A^ and p15^INK4B^ by DNMTi in HCT116 cells. (**a**) Relative methylation levels (see Methods) of *CDKN2A* and *CDKN2B* in DNMT1^+/+^ and DNMT1^−/−^ cells without treatment. (**b**) Methylation levels of *CDKN2A* gene promoter after exposure to DAC and aza-TdC. (**c**) p16^INK4A^ protein expression by DAC versus aza-TdC in DNMT1^−/−^ cells and no expression by both agents in DNMT1^+/+^ cells. (**d**) Methylation levels of *CDKN2B* promoter upon exposure to DAC and aza-TdC. (**e**) Expression of p15^INK4B^ protein by DAC versus aza-TdCyd in DNMT1^−/−^ cells and no expression by both drugs in DNMT1^+/+^ cells. (**f**) Chromatin immunoprecipitation demonstrating modulation of acetylation of histone H3 at *CDKN2A* and *CDKN2B* gene loci by DAC in DNMT1^−/−^ versus DNMT1^+/+^ cells. NTC, no template control. All samples in methylation-specific PCR experiments were assayed in triplicate, with at least three independent experiments performed, and data were shown as the ratio of the average methylated DNA in treated to the control. One representative experiment is shown from at least three independent Chromatin immunoprecipitation assays.

To address the role of activation of tumor suppressors on anticancer cell activity, we investigated the CpG demethylation and re-expression of tumor suppressors by DNMTi in HCT116-*DNMT1* model. *CDKN2A* and *CDKN2B* are silenced partially due to the CpG methylation in HCT116 cells^24,25^. The methylation levels of *CDKN2A* and *CDKN2B* without treatment were comparable in HCT116 *DNMT1*^*−/−*^ and *DNMT1*^+*/*+^ cells, and p16^INK4A^ and p15^INK4B^ proteins were not expressed in *DNMT1*^*−/−*^ as those in *DNMT1*^+*/*+^ cells at baseline (Fig. 3a,c,e), consistent with previous reports^7,26,27^. The methylation levels of *CDKN2A* promoter were reduced in both *DNMT1*^*−/−*^ and *DNMT1*^+*/*+^ cells after exposure to 5 µM decitabine for 72 h (Fig. 3b), whereas p16^INK4A^ was re-expressed in *DNMT1*^*−/−*^ cells only (Fig. 3c and Supplementary Fig. 7c). There was little demethylation in both types of cells after exposure to 5 µM aza-T-dCyd. *CDKN2B* promoter methylation was decreased in both *DNMT1*^+*/*+^ and *DNMT1*^*−/−*^ by decitabine, and p15^INK4B^ protein was re-expressed in *DNMT1*^*−/−*^ cells only (Fig. 3d,e, and Supplementary Fig. 7e). Therefore, decitabine demethylated and reactivated expression of p16^INK4A^ and p15^INK4B^ in *DNMT1*^*−/−*^ cells relative to the demethylation only in *DNMT1*^+*/*+^ cells. The demethylation and re-expression of p16^INK4A^ and p15^INK4B^ were not observed in *DNMT1*^*−/−*^ and DNMT1^+/+^ cells using aza-T-dCyd. In addition, azacitidine at same dose levels and time frame triggered demethylation and re-expression of p16^INK4A^ in HCT116 *DNMT1*^*−/−*^ cells (Supplementary Fig. 2a,b, and 9b).

DNMT1 binds to histone deacetylases in the repressive chromatin complex that mediates the transcriptional repression and gene silencing^6,28,29^. We next examined the acetylation status of histone H3 at the *CDKN2A* and *CDKN2B* gene loci in the HCT116-*DNMT1* pair via chromatin immunoprecipitation. DNA in the tumor suppressor gene promoters bound to the acetylated histone-H3 was increased in *DNMT1*^*−/−*^ cells relative to *DNMT1*^+*/*+^ cells upon exposure to decitabine for 72 h (Fig. 3f and Supplementary Fig. 7f). Similar CDKN2A–histone H3 acetylation status was detected in HCT116 *DNMT1*^*−/−*^ versus *DNMT1*^+*/*+^ cells by azacitidine (Supplementary Figs. 2c and 9c). The data suggest that re-expression of p16^INK4A^ and p15^INK4B^ is linked to an increase in histone-H3 acetylation at the gene loci, in addition to demethylation, in *DNMT1*^*−/−*^ cells. Expression of p16^INK4A^ and p15^INK4B^ was not reactivated in *DNMT1*^+*/*+^ cells without upregulating histone-H3 acetylation although *CDKN2A* and *CDKN2B* genes were demethylated by decitabine and azacitidine. The data suggest that suppression of p16^INK4A^ and p15^INK4B^ expression remain in place in the presence of DNMT1 at 72 h of drug treatment.

### Tumor growth inhibition and modulation of DNMT1 and p21 by DNMT inhibitors in animal models

**Figure 4:**
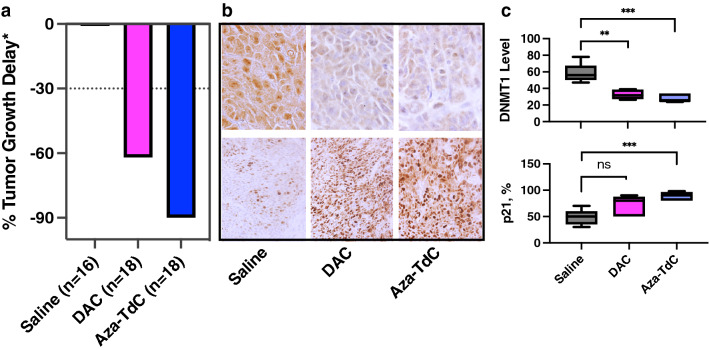
Tumor growth inhibition and modulation of DNMT1 and p21 by DNMTi treatment in HCT116 DNMT1^+/+^ xenograft models. (**a**) Tumor growth delay (see “Methods”) by DAC and aza-TdC relative to the saline treatment. Dotted line indicated 30% of tumor growth inhibition. *The maximal tumor growth delays in DNMT1^+/+^ tumors were observed at day 12 and day 18 of DAC and aza-TdC treatment, respectively. (**b**) Representative expression of DNMT1 and p21 from the tumor samples treated with DAC and aza-TdC. Magnification, ×200. (**c**) Modulation of DNMT1 (upper panel) and p21 (lower panel) by treatment in an average of 5 mouse tumor samples relative to that of saline treatment. **P < 0.01; ***P < 0.001. The solid band in the Box-Whisker plot is mean, top and bottom borders of the plot represent 25% of data greater or less than that value, and the whiskers are maximal and minimal values.

To validate the in vitro findings, mice bearing HCT116 DNMT1^+/+^ xenograft tumors were treated with decitabine and aza-T-dCyd as described in the Materials and Methods. Tumor samples with saline and drug treatment were collected at day 11 of the treatment cycles. The harvested tumors were quartered, in which one piece was formalin-fixed and paraffin-embedded and the cut-sections were used to assess the modulation of DNMT1 and p21. There were significant tumor growth delays, decreasing expression of DNMT1 and upregulating expression of p21 by aza-T-dCyd and decitabine treatment in mice bearing xenograft tumors (Fig. 4a–c). The maximal tumor growth delays in HCT116-*DNMT1*^+*/*+^ model were observed at day 12 and day 18 by decitabine and aza-T-dCyd treatment, respectively. The tumor growth delay and inhibition of expression of DNMT1 and upregulation of p21 were also achieved by decitabine and aza-T-dCyd treatment in bladder PDX model (Supplementary Fig. 3a–c).

### *DNMT1* gene deletion in human malignancies, and its deletion and protein consequence in human colon cancers

**Figure 5:**
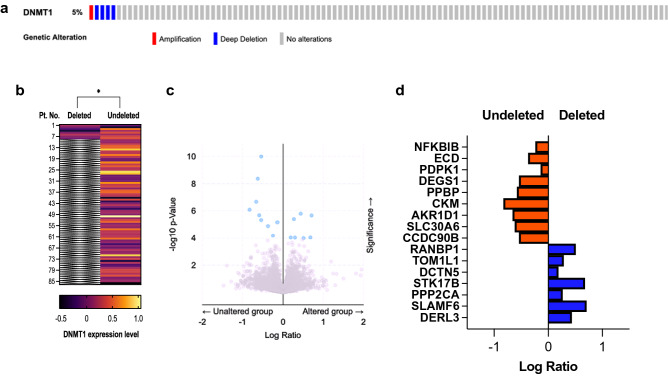
*DNMT1* deletion, its protein consequence and differential expression profile in human colon cancer. (**a**) Oncoprint of *DNMT1* genetic alterations (deep deletion and gene amplification) in 105 cases of colon cancers. (**b**) Heatmap of DNMT1 protein expression in 86 cases without *DNMT1* deletion in relation to the 8 cases with *DNMT1* gene deletion including deep and shallow. Each row represents one patient. *P = 0.017. (**c**) Protein consequence of the genes enriched in the *DNMT1* deleted and undeleted groups shown in volcano plot. (**d**) Bar graph showing top enriched proteins in the deleted (blue bars) and undeleted (orange bars) groups; all *q* values < 0.05.

We analyzed frequency of *DNMT1* gene deletion in human cancers. The deletion was observed in colon cancers, metastatic melanoma and biliary tract cancers, adenoid cystic carcinoma, angiosarcoma and more (Supplementary Fig. 4). Notably, *DNMT1* deletion was not seen in myeloid malignancies. To determine the potential clinical implication of *DNMT1* gene deletion, we investigated *DNMT1* gene deletion with protein consequence in human colon cancer. The deep deletion, and deep and shallow deletion rates were 3.2% (Fig. 5a) and 5.3%, respectively. Expression of DNMT1 protein was significantly lower in 8 cases of the gene-deleted group than undeleted group (86 cases; *P* = 0.017; Fig. 5b). The genes enriched in the *DNMT1* altered group were those implicated in epigenetic regulation, and negative regulation of cell growth and division (Fig. 5c,d). The data suggest that *DNMT1* deletion alteration has specific gene expression profile consequences in human colon cancers.

## Discussion

Our findings demonstrated that DNA damage effects were associated with the anticancer activity of a class of DNMT inhibitors in human solid tumor models in vitro and in vivo. Aza-T-dCyd, decitabine and azacitidine all inhibited the expression of DNMT1 and cell growth, and induced apoptosis and/or cytotoxicity in HCT116 DNMT1^+/+^ colorectal cancer cells. The deletion of *DNMT1* gene alleles markedly attenuated cytotoxic and growth inhibitory effects as well as, of particular, abrogated the DNA damage, cell-cycle arrest and apoptosis/cell death. We validated the findings through CRISPR editing of the *DNMT1* gene in MCF7 breast cancer model. The drugs inhibited expression of DNMT1, cell growth and induced cytotoxicity in DNMT1^+/+^ MCF7 whereas less such effects in its *DNMT1*^*−/−*^ counterpart. Notably, DNMTi generated DNA damage, cell-cycle arrest and apoptosis and cell death in MCF7 *DNMT1*^+*/*+^ but not in DNMT1-disrupted state. Together, DNMT1 is required to the DNA damage effects of anti-DNMT agents. Aza-T-dCyd is more potent than decitabine and/or azacitidine in inhibiting DNMT1 and cell growth and inducing the cytotoxicity and DNA damage effects. This could be related to the addition of 4-thio residue in the chemical structure of aza-T-dCyd relative to decitabine^11^.

**Figure 6:**
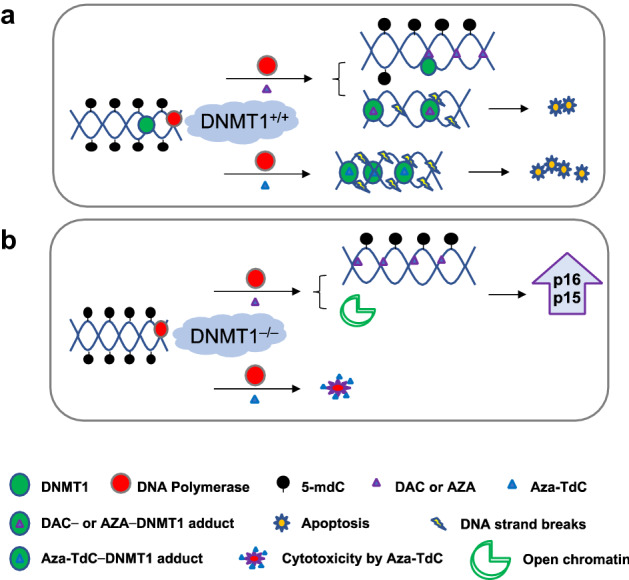
Diagram of the CpG demethylation and DNA damage on the mechanism of DNMTi anticancer activity (DNA damage and apoptosis). (**a**) In DNMT1^+/+^ cells, the incorporation of DAC or AZA into DNA (1) inhibits DNMT1 function and causes DNA demethylation upon replication, and (2) forms the drug-DNMT1 adduct that produces DNA strand breaks and subsequent apoptosis. The incorporation of aza-TdC into DNA forms the drug-DNMT1 adduct that generates severe DNA damage, predominantly triggering apoptosis. (**b**) In DNMT1^−/−^ cells, the incorporation of DAC or AZA into DNA causes demethylation during DNA replication and open chromatin in the absence of DNMT1, leading to the re-expression of tumor suppressors; aza-TdC generates the cytotoxicity. *5-mdC* 5-Methyl- 2'- deoxycytidine.

During DNA replication, cytidine analogs were incorporated into the DNA that covalently bind to DNMT1, forming the DNA–protein adducts which initiate DNA damage and activate the DNA damage response^18,23,30^. By confocal microscopy, we demonstrated that inhibition of DNMT1 concurred with DNA damage response in HCT116 and MCF7 DNMT1^+/+^ cells by both decitabine and aza-T-dCyd at clinically achievable doses. The data reaffirm that decitabine induces DNA damage^18^, and reveal that aza-T-dCyd produces the DNA damage that is more potent than decitabine in DNMT1^+/+^ cells (Fig. 6a,b). The drug treatment did not induce the DNA damage and damage response in DNMT1^−/−^ cells, possibly because of a lack of DNA-DNMT1 adduct formation. As illustrated in Fig. 6a, the incorporation of aza-T-dCyd leads to the formation of more drug-DNMT1 adduct, causing greater DNA damage than decitabine. The presence of 4-thio-residue in the aza-T-dCyd chemical structure may have facilitated the formation of the drug-DNMT1 adduct.

We found an increase in G_2_/M cell-cycle arrest, and apoptosis and cell death in DNMT^+/+^ cells by aza-T-dCyd and decitabine treatment. The G_2_/M arrest in DNMT^+/+^ cells was associated with an upregulation of p21 and activation of Chk1-Ser345^31–33^. The higher basal level of γH2AX in DNMT1^−/−^ cells was likely due to that *DNMT1* knockdown led to the genomic instability, disrupting the check and balance of cellular activity, causing higher basal level of expression of the effectors implicated in the DNA damage response signaling and cell-cycle checkpoints such as γH2AX, p-CHK1, p21 and p-CDC-2^34,35^. We validated the results in vivo, with aza-T-dCyd more potent in inhibiting the growth of HCT116 DNMT^+/+^ xenograft and bladder PDX tumors. DNMT1 was inhibited and p21 was up-modulated in these tumor samples. The increase in p21 was inversely associated with the tumor growth inhibition of anti-DNMT agents in the xenograft and PDX tumors^36^.

Through examining the methylation status of the tumor suppressor gene promoters following DNMTi exposure, we found the demethylation of *CDKN2A* and/or *CDKN2B* genes in DNMT1^+/+^ and DNMT1^−/−^ cells by decitabine and azacitidine. Expression of p16^INK4A^ and p15^INK4B^ proteins was, however, reactivated in DNMT1^−/−^ cells only. As the levels of demethylation in DNMT1^+/+^ and DNMT1^−/−^ cells were roughly comparable, and we further investigated the implication of DNMT1 in the repression of the tumor suppressors at their associated chromatin in HCT116-*DNMT1* model^28,29^. The DNA in *CDKN2A* and *CDKN2B* promoters were connected to an enhanced acetylation of histone-H3 following exposure to decitabine and/or azacitidine, indicative of more assessable chromatin state in the absence of DNMT1. Hence, the reactivation of expression of tumor suppressors is ascribed to the fact that decitabine and azacitidine were more effective for demethylating DNA and generating accessible chromatin when DNMT1 was knocked down^28,29,37^. In the presence of DNMT1, decitabine and azacitidine demethylated DNA, but did not increase the level of histone-H3 acetylation at *CDKN2A* and *CDKN2B* loci nor induced the expression of p16^INK4A^ and p15^INK4B^. Therefore, the gene repression was still in place in the presence of DNMT1 despite demethylation (Fig. 6a,b). Aza-T-dCyd (5 µM) did not significantly demethylate nor reactivate the expression of the tumor suppressors. This is likely due to that aza-T-dCyd predominantly eliminated the cells with the severe DNA damage by undergoing apoptosis and cell death. However, more cells with lesser degree of DNA damage induced by decitabine and/or azacitidine could continue to replicate during cell division and causes subsequent demethylation and tumor suppressor reactivation. This phenomenon was described as that decitabine and azacitidine demethylated promoter DNA slowly^38^. The induction of p16^INK4A^ expression by DNMTi could occur in the presence of DNMT1 with the prolonged drug exposure in cancer cells^39^ and in human tumor samples with several cycles of DNMTi treatment^40^.

Moreover, through the genomic analyses in human colon cancers and other malignancies, we found that *DNMT1* gene deletion had reduced protein expression and exhibited specific gene expression profiles in colon cancers. *DNMT1* gene deletion and lack of or low protein expression may serve as an exclusion criterion from DNMTi therapy according to the in vitro and in vivo findings. The gene deletion was also seen in other cancer types but not in myeloid malignancies.

In conclusion, it was the DNA damage effects in the presence of DNMT1 without re-expression of tumor suppressors that were responsible for the anticancer activity of prototypical and novel DNMT inhibitors. In contrast, the reactivation of expression of p16^INK4A^ and p15^INK4B^ was implicated in the reduced cytotoxicity and growth inhibition and a lack of DNA damage effects when *DNMT1* gene was deleted or disrupted. The findings add new knowledge and understanding to the DNA demethylation mediated by DNMTi and provide rationale to accelerate appropriate application of a class of the agents that target the DNA methyltransferase for cancer treatment.

## Methods

### Cell lines and drugs

Decitabine and aza-T-dCyd, and azacitidine were obtained from the Developmental Therapeutics Program, Division of Cancer Treatment and Diagnosis, National Cancer Institute, and purchased from Millipore-Sigma. Human colorectal cancer HCT116 cells (DNMT^+/+^) and its isogenic DNMT1 knockout (DNMT1^−/−^, DNMT1-deficient) counterpart were obtained from Horizon Discovery, which was recommended by Dr. Bert Vogelstein (Sidney Kimmel Comprehensive Cancer Center of the Johns and Hopkins University, Baltimore, Maryland). The HCT116 DNMT1^−/−^ cell line was generated by homozygous deletion of DNMT1 gene (DNMT1-Δexons3-5/Δexons3-5)^26^ and cultured in RPMI-1640 supplemented with 10% of FBS and 2 mM of l-glutamine. MCF7 cells was obtained from the Tumor/Cell Line Repository, Division of Cancer Treatment and Diagnosis, National Cancer Institute (Frederick, MD).

### *DNMT1* gene disruption in MCF7 cells by CRISPR method

The targeted *DNMT1* gene editing in MCF7 cells was performed using a CRISPR-CAS9 method per manufacturer’s instructions (OriGene, Rockville, MD). Briefly, cells were co-transfected with the pCAS9-guide vector containing a DNMT1 target sequence (guide RNA or gRNA) and the DNA donor vector containing the homologous arms and a functional cassette (GFP and puromycin resistance gene). After transfection, the CAS9 enzyme cut double strand breaks in the chromosome and the DNA donor vector was integrated into the genome replacing the target gene through homologous recombination. Briefly, 200,000 cells were seeded in 24 well plate overnight. One µM of the gRNA vector or 1 µM donor vector or scrambled control and the donor vector were mixed with 100 µl opti-MEM. The transfected cells were cultured for a month before puromycin selection. Puromycin was added to the cells for 5 days and individual cell colonies were isolated. The knockout effect was examined by Western blot with DNMT1 antibody (clone EPR3521(2); Abcam) in the puromycin-selected cells.

### MTT assay

Cells (0.4 × 10^4^ cells/well) in triplicates were seeded in 96 well plates. They were treated with increasing concentrations of DNMT inhibitors for 96 h, and cell viability was measured by MTT (3-[4,5-dimethylthiazol-2-yl]-2,5-diphenyltetrazolium bromide) assay. Afterwards, MTT solution was added to each well, and the plates were incubated for additional 4 h at 37 °C and followed by incubation at 37 °C overnight after addition of 10% SDS/0.01 M HCl solution. The formazan dye formed by the viable cells was quantified by measuring the absorbance of the dye solution at 590 nM.

### Annexin-V/propidium iodide apoptosis assay

Cells were treated with increasing concentrations of DNMT inhibitors. After treatment, cells were labeled with annexin-V-FITC and propidium iodide (PI)-PE (R&D Systems) and analyzed with a FACSCanto II flow cytometer (Becton Dickinson) using the BD FACSDiva™ Software.

### Clonogenic cell survival assay

The test has been described in detail previously^41^. Briefly, 150,000 cells were treated with increasing concentrations of DNMTi for 48 h. After washing, 2,000 cells/well in triplicates were plated in 6 wells plates for 8–14 days. They were fixed using 1 ml of fixative buffer (10% methanol plus 10% acetic acid glacial) and stained with 1 ml of 0.1% crystal violet. The colonies were washed with dH2O, dried at room temperature and counted using Automated Colony Counter (AccuCount™ 1000 from BioLogics Inc.).

### Cell cycle analysis

Cell cycle analysis has been described previously^31^. In brief, cells were treated with DNMTi. After 24 h of treatment, they were detached, washed and fixed in cold 70% ethanol overnight. After washing, cells were stained with 1 ml of propidium iodide solution (50 µg/ml propidium iodide) supplemented with 50 µl RNase A (50 mg/ml) for 1 h at 37 °C and analyzed with a FACSCanto II Cell Cytometer (BD Becton Dickinson Biosciences, Franklin Lakes, NJ). At least 20,000 cells were collected, and the cell cycle profiles were calculated using the FlowJo software (Becton Dickinson).

### Western blotting

Western blotting method has been described previously^41^. Briefly, 50 µM of protein were electrophoresed on 7.5% or 4–20% SDS–polyacrylamide gels (Bio-Rad, Hercules, CA, USA). The primary antibodies used for Western blot analysis were: DNMT1 (1:1000; Abcam), p16 (1:1000; Abcam), p15 (1:1000; ThermoFisher Scientific) and pHH3-S10 (1:5000; Abcam (1:5000; Abcam), γH2AX (1:1000; Millipore Sigma); p21 (1:1000; Cell Signaling Technology), pCHK1-Ser345 (1:500; Cell Signaling Technology), pCDC2-Y15 (1:100; Santa Cruz) and β-actin (1:30,000; Sigma Aldrich). Densitometry and quantitative analysis of images were performed using NIH Image J software.

### Confocal microscopy

For detection of DNMT1 and γH2AX in situ following drug treatment, cells were suspended in PBS and added to the EZ cytofunnel chamber to prepare cytospins. They were centrifuged at 600 rpm for 10 min, with approximately 200,000 cells per slide, which were then fixed in 4% paraformaldehyde for 15 min at room temperature; followed by permeabilizing with 0.1% Triton-X100 PBS solution at RT for 10 min. For staining, γH2AX antibody in 1:300 dilution (Millipore Sigma) and DNMT1 antibody in 1:500 dilution (Abcam) was incubated overnight at 4 °C. Antibody attachment was reported using a conjugate reporter stain of either anti-mouse Alexa Fluor 555 or anti-rabbit Alexa Fluor 488. Image capture was conducted on a Carl Zeiss LSM 710 NLO confocal microscope. The areas of cells were selected based on DAPI staining, excluding thick cellular areas with overlapping cells, areas with artifacts, or poor staining. Average fluorescence intensity was calculated using ZEN 2009 Image Pro Premier (ZEISS).

### Real-time methylation-specific PCR

DNA was extracted using PureLink™ Genomic DNA Mini Kit (ThermoFisher Scientific) according to the manufacturer’s instructions. Methylation-specific PCR (MSP) was performed as described by Herman et al.^42^. Five hundred nanograms of DNA was subjected to bisulphite conversion of cytosine to thymine in DNA with the EpiTect Bisulfite kit (Qiagen). Real-time PCR was performed using 7900HT Applied Biosystems, with the Syber Green PCR Master Mix as the intercalating dye. The reaction mixtures consisted of 25 ng of bisulphite-modified template, 200 nmol/l of each primer in a final volume of 15 µl. CtCG represents the threshold cycle for the methylated CG reaction, and CtTG represents the threshold cycle of the unmethylated reaction. Relative levels of methylated DNA (percentage) in each sample were calculated according to the equation, Cmeth = 100/[1 + 2(CtCG − CtTG)]%^43^. The CpGenome universal methylated and unmethylated DNA (Millipore Sigma) were used as positive and negative controls, respectively. The primer sets to amplify methylated and unmethylated CDKN2A and CDKN2B genes had been described by Herman et al.^42^.

### Chromatin immunoprecipitation (ChIP)

Cells were treated with decitabine and azacitidine or vehicle in 6-well plates for 72 h. The ChIP assay was performed using High Sensitivity ChIP Kit (Abcam) according to the manufacturer’s instructions. After washing, protein and DNA complexes in cells were crossed linked in 1% formaldehyde culture medium at room temperature for 10 min. DNA in the nuclear fraction was sheared by sonication and the supernatant containing the protein-DNA complex were incubated with an antibody (Abcam) to acetyl-histone H3 (1 µg) in each well for 1 h. Following decrosslinking and proteinase K treatment, the antibody-precipitated DNA was purified. The DNA was amplified using PCR with T100 Thermal Cycler (Bio-Rad), and PCR product was analyzed by 4% agarose gel. The primers and PCR conditions to amplify the CDKN2A and CDKN2B promoters were described previously^42^.

### In vivo treatment of xenograft and PDX tumors, and immunohistochemistry

All methods were reported in accordance with Animal Research: Reporting of In Vivo Experiments (ARRIVE) guidelines. Female athymic nude mice (nu/nu NCr; Animal Production Program, National Cancer Institute at Frederick, Frederick, MD) were implanted by subcutaneous injection of a cell suspension of human cancer cells into the flank tissues as described previously^44^. The mice were assigned to the treatment groups for each model when the tumors reached a median tumor staging size of 150 mg in weight. They were subsequently treated with decitabine at 0.75 mg/kg, IP and aza-T-dCyd at 2 mg/kg or 1 mg/kg, IP or saline in mice bearing DNMT1^+/+^ HCT116 xenograft and bladder PDX (BL0382) tumors. The mice were dosed daily for 5 days, rest for 2 days, and repeat dosing for 5 days and rest for 9 days in a 21-day cycle for 2–3 cycles except short decitabine dosing duration in PDX models due to the toxicity. Tumors were monitored by bidirectional caliper measurements, and the tumor weights were calculated as tumor weight (mg) = (tumor length in mm × tumor width in mm^2^)/2. Tumor growth delay was calculated by [(T − C)/C]. The National Cancer Institute Frederick National Laboratory for Cancer Research is accredited by AAALAC International and follows the Public Health Service Policy for the Care and Use of Laboratory Animals. All animal experiments were approved by Animal Care and Use Committee of the National Cancer Institute and conducted in accordance with the institutional guidelines and regulations.

Immunohistochemistry on the formalin-fixed and paraffin-embedded sections was described previously^45^, and used to examine DNMT1 and p21 expression in in vivo tumor samples. The primary antibodies used were: DNMT1 (clone EPR3521(2); 1:500; Abcam) and p21 (clone 12D1; 1:50; Cell Signaling Technology). Areas of tumor staining were analyzed with the assistance of a digital imaging system (DAKO, Carpinteria, CA) reporting the intensity and percentage of staining for DNMT1 to determine the staining Index (SI). It was calculated as the percentage multiplied by intensity of staining (after subtracting the tissue readout of the corresponding negative control) divided by 100 (SI = intensity × percentage/100)^46^. p21 was reported as the percentage of tumor staining as described previously^47^.

### Genomic analysis of *DNMT1* gene deletion in human cancers

DNMT1 gene alterations were analyzed in 68,088 cases of human malignancies from the data at cBioPortal for Cancer Genomics^48,49^. We also analyzed the *DNMT1* deletion (deep and shallow) with protein expression status in 105 cases of human colon tumors. The data were lastly accessed on September 10, 2022.

### Statistical analysis

All numeric data were derived from the experiment(s) assayed in triplicate with at least three independent experiments performed and shown as means ± standard deviation (SD). Statistical analyses were performed using GraphPad Prism version 9.0. with unpaired or paired *t*-test (two-sided) as appropriate. A *P* value less than 0.05 was prespecified to be statistically significant.

### Ethics approval

Clinical material at cBioportal is deidentified and ethics approval is not required. All animal studies were conducted per an approved animal care and use committee protocol in accordance with the procedures outlined in the Guide for Care and Use of Laboratory Animals.

## Supplementary Information

Supplementary Information.

## Data Availability

The dataset generated and/or analyzed during the current study are available from the corresponding author on reasonable request.
