# Supplementary material for: DNA damage, demethylation and anticancer activity of DNA methyltransferase (DNMT) inhibitors
Source: Sci Rep. 2023 Apr 12;13:5964. doi: 10.1038/s41598-023-32509-4 (PMC10097729; doi:10.1038/s41598-023-32509-4)
Supplement: Supplementary file 1 — Supplementary Information. [file 41598_2023_32509_MOESM1_ESM.pdf]

Supplementary Information

DNA damage, demethylation and anticancer activity of DNA methyltransferase (DNMT) inhibitors

Angelo B. A. Laranjeira, Melinda G. Hollingshead, Dat Nguyen, Robert J. Kinders, Michael Difilippantonio, James H. Doroshow, and Sherry X. Yang

Supplementary Table: Quantitative results of Western blots in Fig. 2f.

Supplementary Figure 1: Effects of DNMTi on DNMT1, growth, cell-cycle and apoptosis.

Supplementary Figure 2: Demethylation of *CDKN2A* gene and acetylation of histone H3, and re-expression of $p16^{INK4A}$ by AZA in HCT116 cells.

Supplementary Figure 3: Tumor growth delay and modulation of DNMT1 and p21 by drug treatment in bladder PDX tumors.

Supplementary Figure 4: Distribution of *DNMT1* gene alterations in 68088 cases of human cancers.

Supplementary Figure 5: Full length Western blot images in Figure 1.

Supplementary Figure 6: Full length Western blot images in Figure 2f.

Supplementary Figure 7: Full length Western blot and agarose gel images in Figure 3.

Supplementary Figure 8: Full length Western blot images in Supplementary Figure 1.

Supplementary Figure 9: Full length Western blot and agarose gel images in Supplementary Figure 2.

Table. **Quantitative results of Western blots in Fig. 2f**.

	HCT116 DNMT1$^{+/+}$						HCT116 DNMT1$^{-/-}$					
Drug concentration (μM)	0.00	0.50	5.00	0.00	0.50	5.00	0.00	0.50	5.00	0.00	0.50	5.00
DAC	-	+		-	-	-	-	+	+	-	-	-
Aza-TdC	-	-	-	-	+	+	-	-	-	-	+	+
Ratio of protein level to β-actin												
γH2AX	0.21	0.24	0.23	0.35	0.45	0.41	0.79	0.86	0.85	0.91	0.83	0.63
pCHK1-S345	0.20	0.29	0.30	0.09	0.33	0.31	0.58	0.68	0.73	0.40	0.42	0.42
p21	0.12	0.16	0.13	0.03	0.41	0.42	0.40	0.60	0.56	0.30	0.63	0.49
pCDC2-Y15	0.07	0.16	0.13	0.11	0.23	0.29	0.30	0.32	0.33	0.33	0.38	0.33

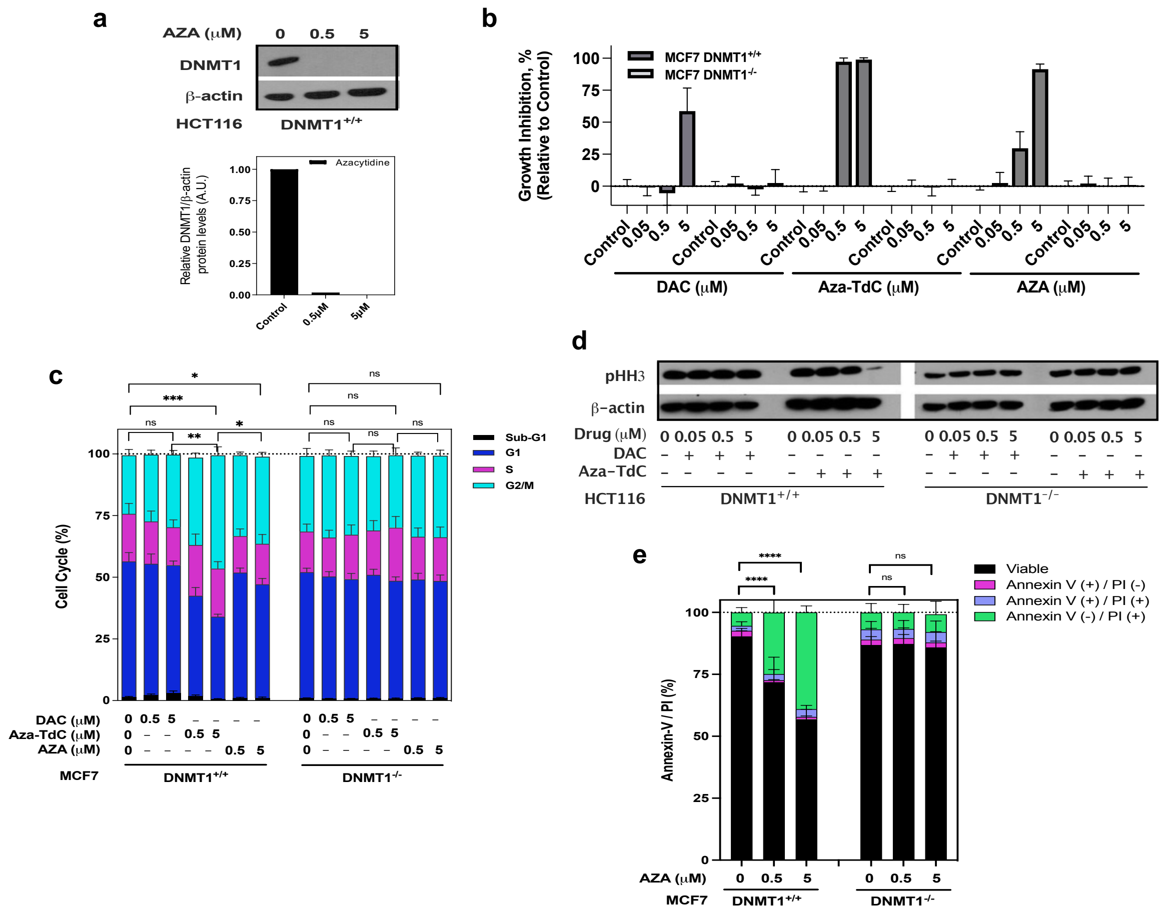

Supplementary Figure 1: Effects of DNMTi on DNMT1, growth, cell-cycle and apoptosis. **a** Inhibition of DNMT1 protein by AZA in HCT116 DNMT1$^{+/+}$ cells. **b** Colony formation inhibition of the MCF7 pair by DAC, aza-TdC and AZA. **c** G_2/M cell-cycle arrest induced by DNMTi in MCF7 cells. **d** Histone H3 phosphorylation (pHH3-S10) after DAC and aza-TdC treatment for 24h in HCT116 pair. **e** Induction of apoptosis and cell death upon exposure to AZA for 96h via Annexin-V/PI apoptosis assay in the MCF7 pair. All samples in the clonogenic, cell-cycle and annexin-V/PI apoptosis tests were assayed in triplicate, with at least three independent experiments performed, and data were shown as mean ± SD. **P*<0.05; ***P*<0.01; ****P*<0.001; *****P*<0.0001; and ns, not significant. PI, propidium iodide. One representative experiment is shown from at least three independent experiments performed for other assays.

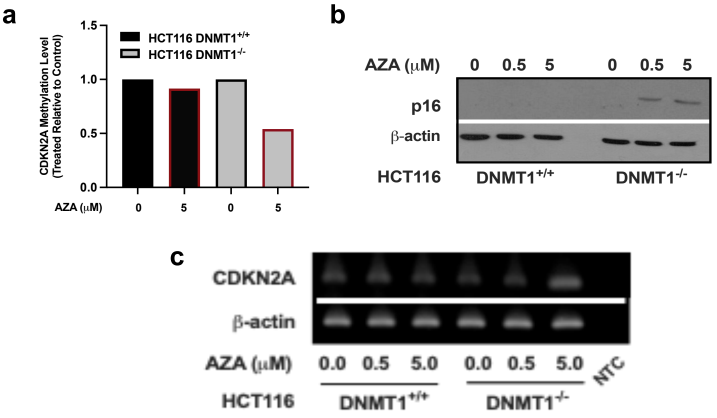

Supplementary Figure 2: Demethylation of *CDKN2A* gene and acetylation of histone H3, and re-expression of p16^{INK4A} by AZA in HCT116 cells. **a** Methylation levels of *CDKN2A* gene promoter after exposure to AZA. **b** p16^{INK4A} protein expression in DNMT1$^{-/-}$ *versus* DNMT1$^{+/+}$ cells by AZA. **c** Chromatin immunoprecipitation showing modulation of acetylation levels of histone H3 at *CDKN2A* gene locus by AZA in DNMT1$^{-/-}$ versus DNMT1$^{+/+}$ models. NTC, no template control.

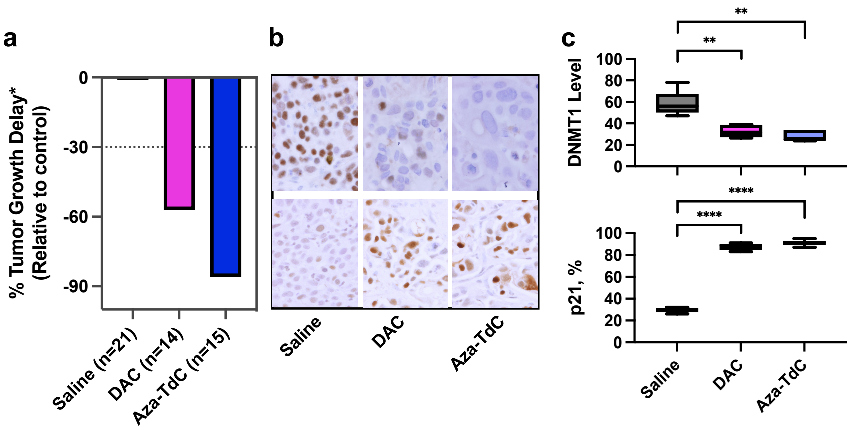

Supplementary Figure 3: Tumor growth delay and modulation of DNMT1 and p21 by treatment in bladder PDX tumors. **a** Tumor growth delay (see Methods) by DAC and aza-TdC treatment. Dotted line indicated 30% of tumor growth delay. *The maximal tumor growth delays in DNMT1$^{+/+}$ PDX tumors were observed at day 25 and day 56 of decitabine and aza-TdC treatment, respectively**.** **b** Representative expression of DNMT1 (upper panel) and p21 (lower panel) from the tumor samples treated with DAC and aza-TdC. Magnification, X200. **c** Modulation of DNMT1 and p21 by treatment (n=4 per group). ***P*<0.01, *****P*<0.0001. Note: the solid band in the Box-Whisker plot is mean, top and bottom borders of the plot represent 25% of data greater or less than that value, and the whiskers are maximal and minimal values.

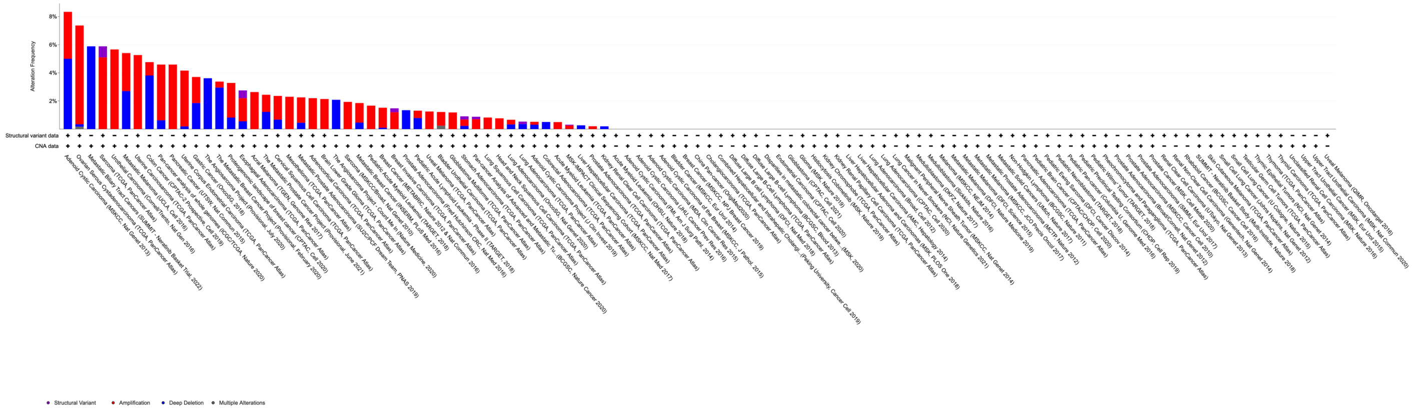

Supplementary Figure 4: Distribution of *DNMT1* gene alterations in 68088 cases of human cancers. Blue, deep deletion; Red, amplification; Purple, structural variant; Dark green, multiple alterations.

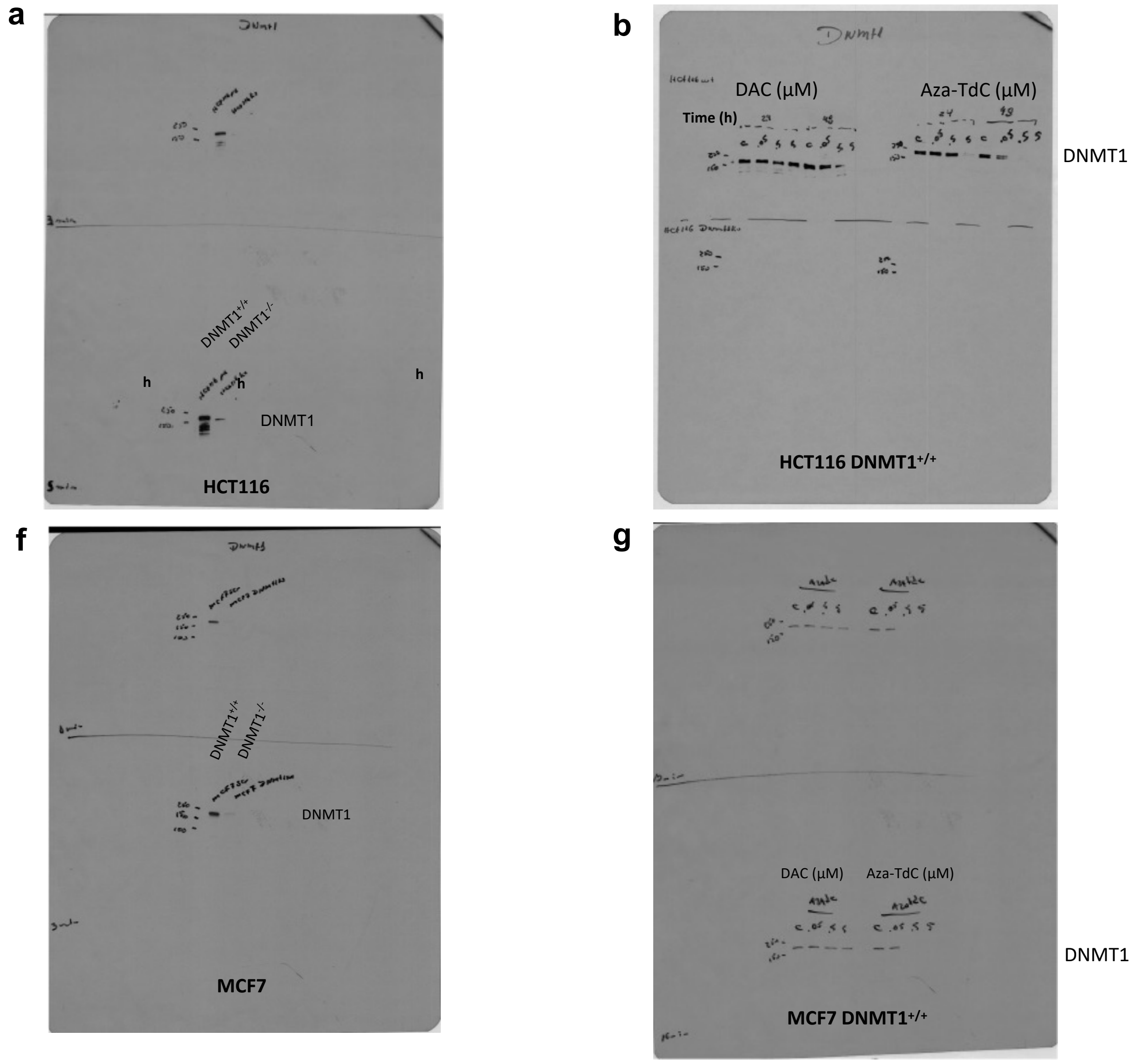

Supplementary Figure 5: Full length Western blot images in Figure 1. **a** Full length blot images in Figure 1a. **b** Full length blot images in Figure 1b. **f** Full length blot images in Figure 1f. **g** Full length blot images in Figure 1g.

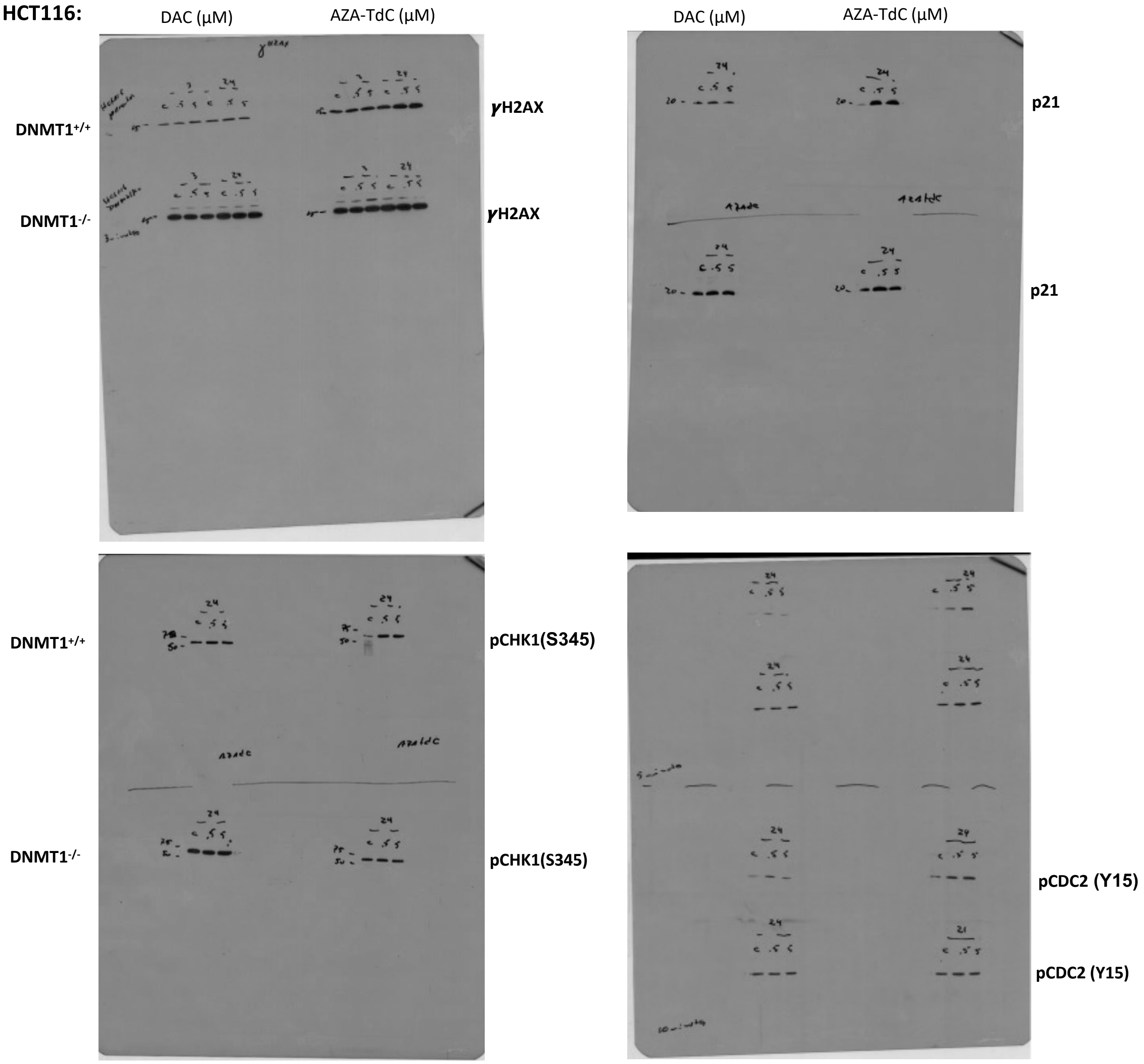

Supplementary figure 6: Full length Western blot images in Figure 2e.

c

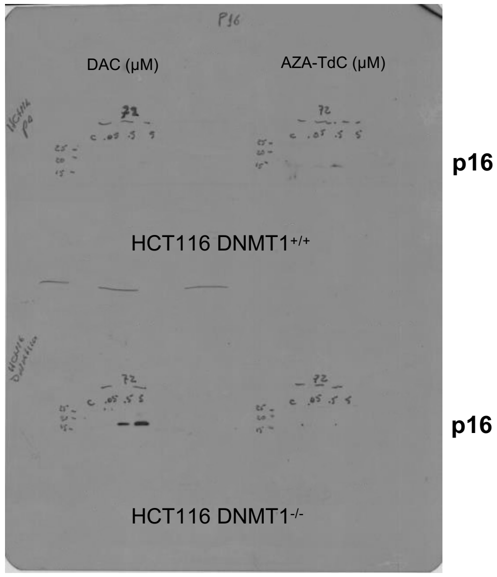

e

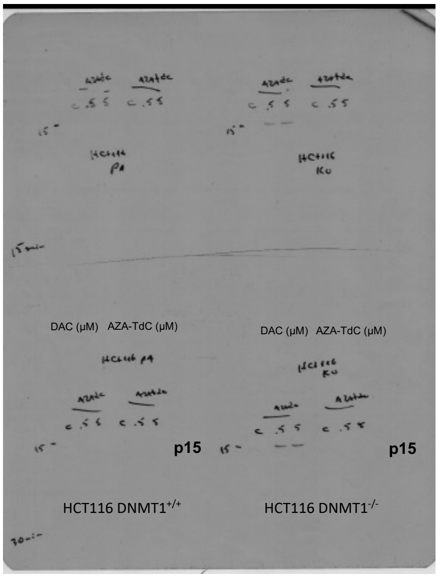

f

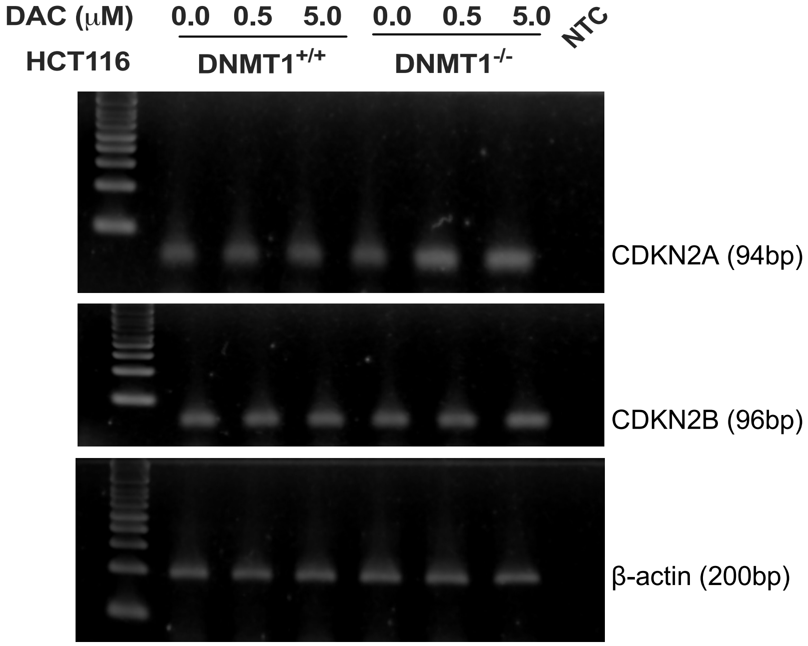

Supplementary Figure 7: Full length Western blot and agarose gel images in Figure 3. **c** Full length Western blot images in Figure 3c. **e** Full length Western blot images in Figure 3e. **f** Unprocessed agarose gel images in Figure 3f.

a

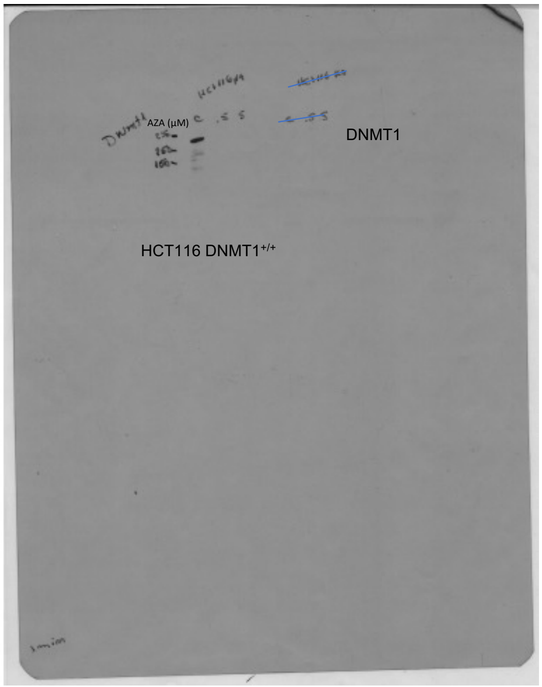

d

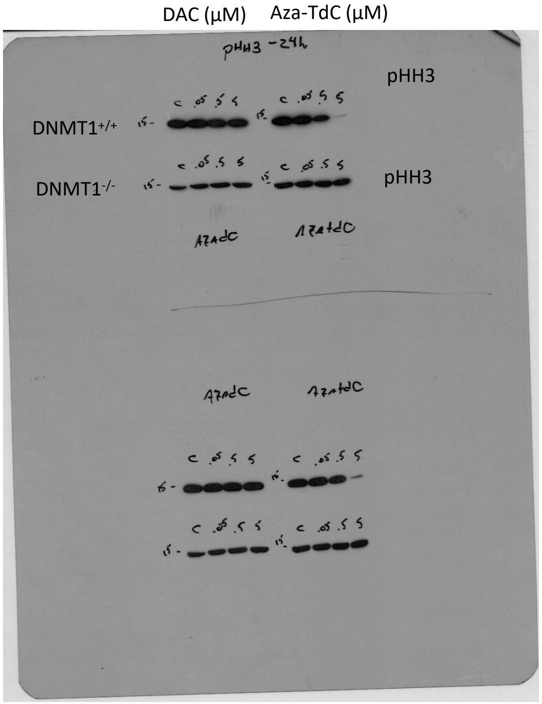

Supplementary Figure 8: Full length Western blot images in Supplementary Figure 1. **a** Full length Western blot images in Supplementary Figure 1a. **d** Full length Western blot images in Supplementary Figure 1d.

b

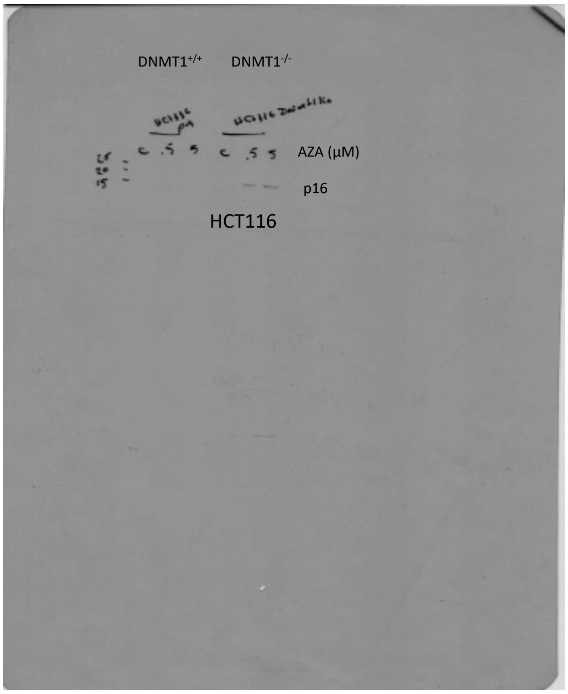

c

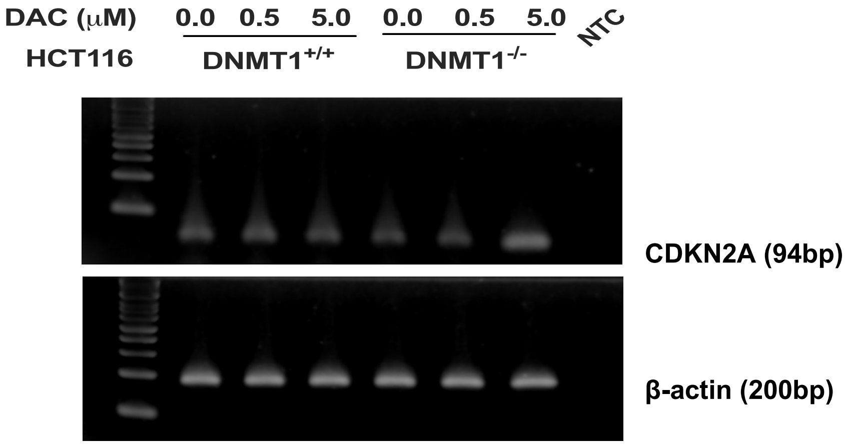

Supplementary Figure 9: Full length Western blot and agarose gel images in Supplementary Figure 2. **b** Full length Western blot images in Supplementary Figure 2b. **c** Original agarose gel images in Supplementary Figure 2c.
